# A comparison of thrombosis in total knee arthroplasty with and without a tourniquet: a meta-analysis of randomized controlled trials

**DOI:** 10.1186/s13018-021-02366-w

**Published:** 2021-06-25

**Authors:** Jia Xie, Hao Yu, Fangyuan Wang, Juehua Jing, Jun Li

**Affiliations:** Department of Orthopedics, The Second Affiliated Hospital of Anhui Medical University, Hefei, 230601 Anhui China

**Keywords:** Total knee arthroplasty, Tourniquet, Thrombosis, Meta-analysis, Complication

## Abstract

**Background:**

Tourniquets are widely used in total knee arthroplasty (TKA), but the issue of their safety remains controversial. Previous studies have focused on TKA blood loss, duration of surgery, and hemostatic drugs. The purpose of this meta-analysis was to analyze the effect of tourniquet use on postoperative deep venous thrombosis (DVT).

**Methods:**

PubMed, SCOPUS, Web of Science, Embase, and the Cochrane Library were searched for randomized clinical trials published before April 17, 2020, that compared the effect of tourniquet use on postoperative DVT, knee circumference, D-dimers, and pain measured using the visual analog scale (VAS).

**Results:**

Fourteen clinical trials that included 1321 unique participants were included in the meta-analysis. Among the total, 721 and 600 participants were randomized to the tourniquet and non-tourniquet groups, respectively. The incidence of postoperative thrombosis in the tourniquet group was significantly higher than in the non-tourniquet group (RR 2.30, 95% CI 1.51–3.49, *P* < 0.0001, *I*^2^ = 0%). On the 1st, 3rd, and 5th to 21st days, and 3 to 6 weeks after surgery, the knee circumference difference of the tourniquet group was significantly larger than that of the non-tourniquet group (*P* < 0.05). However, 4 to 6 months after the surgery, no significant difference in knee circumference was found between the two groups (MD 0.14, 95% CI −0.02–0.31, *P* = 0.09, *I*^2^ = 0%). The VAS score of the tourniquet group was higher than the non-tourniquet group on the 3rd and 5th days after surgery (*P* < 0.05). However, this difference was not significant (MD 0.31, 95% CI −0.05–0.66, *P* = 0.09, *I*^2^ = 89%).

**Conclusion:**

Results of this meta-analysis indicate that tourniquet application could increase the incidence of postoperative DVT and aggravate postoperative pain and swelling in the short term.

**Level of evidence:**

Level III

## Introduction

Total knee arthroplasty (TKA) is a common surgical procedure for painful arthritis of the knee. Its main purpose is to relieve knee pain and restore joint stability and function. TKA has been proven a successful surgical procedure that could restore a significant degree of function in arthritic knees in most cases [1, 2]. Patient satisfaction with the implantation of total knee endoprostheses was about 81.4% [3]. Pneumatic tourniquets have been widely used in various orthopedic surgeries since its introduction by Harvey Cushing in 1904. In TKA, tourniquets have been used for more than a century, aiming to provide a clear surgical field of vision to shorten the operation time and improve the accuracy of the procedure [4, 5]. Although tourniquets are widely used, the complications caused by its alteration of normal physiological state should not be ignored. Examples of these are compartment syndrome, DVT, skin necrosis and neurological complications, and even permanent damage and loss of limb function [6–9]. Tourniquet use is considered as an important cause of DVT [10]. However, several previous meta-analyses have shown no difference in the effects of TKA with and without tourniquet use on postoperative DVT [11, 12], but others believe that the incidence of DVT in the tourniquet group was higher than that in the non-tourniquet group [13, 14]. Compared with the previous meta-analysis, this study included recent studies different from the ones analyzed before, and a few comparisons of knee circumference not seen in previous meta-analyses were also made.

Studies on tourniquet use in TKA are numerous and varied. Conducting a meta-analysis seems to be a good and comprehensive method to analyze these data. The purpose of this study was to evaluate the effect of tourniquet use during unilateral TKA on postoperative DVT, so as to provide guidance for clinical practice.

## Material and methods

This meta-analysis was performed in accordance with the guidelines listed in the Preferred Reporting Items for Systematic Reviews and Meta-Analyses (PRISMA).

### Search strategy

We systematically searched PubMed, SCOPUS, Web of Science, Embase, and the Cochrane Library for randomized control trials published before April 17. The search was performed using the PICO model [15]. The key words used were “total knee arthroplasty,” “total knee replacement,” “TKA,” “TKR,” “tourniquets,” “tourniquet,” “thrombosis,” “thromboses,” “thrombus,” “blood clot,” and “blood clots.” There was no language or study type restriction, and we manually searched the reference lists of included studies. All articles were imported into a literature management software and screened for duplicates. Two reviewers independently selected the title and abstract of each article and had discussions to resolve the differences. A third reviewer resolved any remaining disagreements.

### Inclusion and exclusion criteria

Studies included in this meta-analysis met the following inclusion criteria: (1) randomized controlled trials (RCT), (2) the study compared TKA with and without tourniquet use, (3) osteoarthritis was managed with TKA, and (4) the primary outcome was DVT and the secondary outcomes were knee circumference, D-dimers, and pain.

The exclusion criteria were as follows: (1) bilateral knee replacement, (2) involved other types of surgery (knee arthroscopy, hemiarthroplasty, fracture fixation, etc.), and (3) any non-human studies, regardless of the type or size of prosthesis, anesthesia, and postoperative care. The date of follow-up examinations in individual studies usually varies, so similar follow-up examination time points were systematically combined for analysis (e.g., second to third postoperative day).

### Quality assessment

We evaluated the quality of the explicit eligibility criteria, similarity of baseline characteristics, and as well as the revised Jadad scale of the included studies [16]. We resolved disagreements by discussion or adjudication by another reviewer. The revised Jadad scale has a maximum score of 7 points assessed with the following items: (1) random sequence generation (0–2 points), (2) allocation concealment (0–2 points), (3) double-blind design (0–2 points), and (4) the analysis and reasons for withdrawals and dropouts (0–1 point). Studies with a score of at least 4 are considered good quality, and poor-quality studies have a score of less than 3.

### Statistical analysis

A meta-analysis was performed with the studies using the Review Manager Database (RevMan version 5.3, Cochrane Collaboration, Copenhagen, Denmark, 2014). Mean differences (MD) were used to weigh the effect size for continuous outcomes, and relative risks (RR) were used for dichotomous outcomes. Funnel plots were used to examine publication bias. We assessed the presence of statistical heterogeneity with the use of a standard Chi square test and the value of *I*^2^. It could be considered suggestive of statistical heterogeneity, prompting a random effects modeling estimate if *P* < 0.1 and *I*^2^ > 50%. Otherwise, we used a fixed effects approach.

## Result

**Fig. 1:**
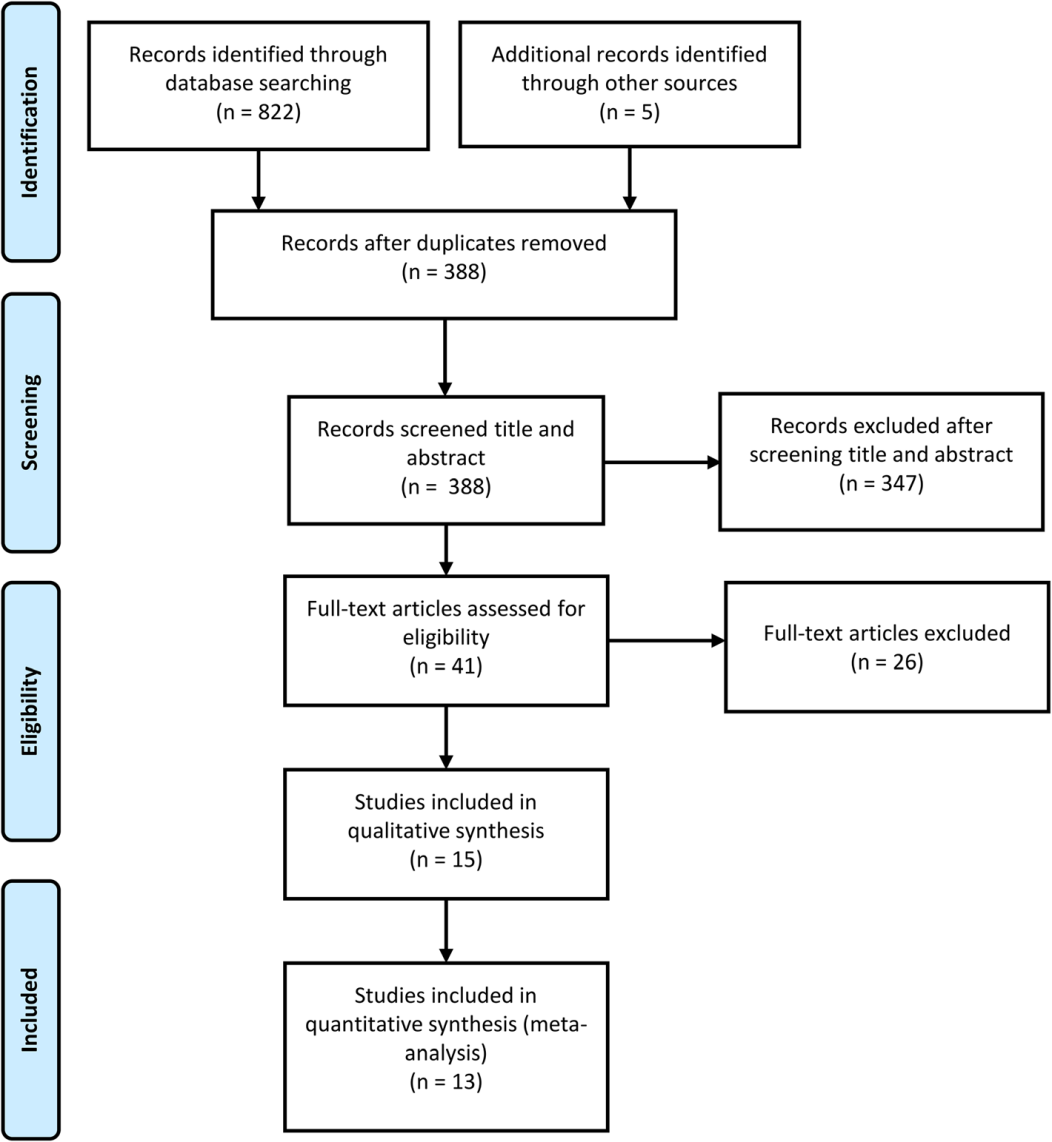
Flowchart of search strategy

A total of 822 articles were retrieved from the database using our search strategy, and 5 articles were obtained from other review references. After removing 439 duplicate articles, 388 articles were screened. Among these, 347 articles were excluded after reading the titles and abstracts, and 26 articles were excluded after reading the full text. This screening resulted in 15 articles enrolling a total of 1400 patients, all of which compared TKA with and without tourniquet use. After quality evaluation, two studies [17, 18] were excluded due to poor literature quality. Finally, 13 RCTs [19–31], enrolling a total of 1321 patients, were included in the meta-analysis; 721 participants were randomized to the tourniquet group, while 600 were randomized to the non-tourniquet group (Fig. 1). The data extracted from the included studies (age, tourniquet pressure, and operating time) are summarized in Table 1. In the included studies, tourniquets of varying pressures were used, and most of them were deflated after the incision was closed. The type of anesthesia and anticoagulant regimens used in all operations were dependent on the surgeon’s experience and the patient’s needs.

**Table 1:** Characteristics of the included studies

Author	Year	Age (T/N T)	Number of patients	T/N T	Tourniquet pressure	Operation time (min)	DVT	K-C	D-d	Pain
Abdelsalam	1995	72.0/74.0	80	40/40	Twice SBP	<90	✓			✓
Wakankar	1999	72.5/71.8	77	37/40	Twice SBP	NA	✓	✓		✓
Aglietti	2000	70.0/68.0	20	10/10	0.8 bar	90.0			✓	
Vandenbussche	2002	72.5/68.5	80	40/40	350 mmHg	151.0	✓			✓
Wauke	2002	63.2/61.4	37	19/18	SBP+100 mmHg	75.1	✓			
Li	2008	71.0/70.0	80	40/40	SBP+100 mmHg	73.0		✓		
Zhang	2010	72.0/71.0	60	30/30	SBP+100 mmHg	<90	✓			
Yin	2011	63.1/61.9	39	20/19	NA	NA			✓	
Yin	2012	68.9/67.1	40	20/20	NA	NA			✓	
Ejaz	2014	68.0/68.0	92	33/31	250 mmHg	70.0	✓			✓
Mori	2016	72.8/74.6	103	51/52	250 mmHg	63.6	✓			
Zhang	2016	63.2/65.2	230	84/82	SBP+13.3 kPa	81.7	✓			✓
Zhou	2017	66.8/69.1	150	72/68	NA	77.2	✓	✓		✓
Wu	2018	67.6/68.1	112	50/50	250 mmHg	67.2	✓	✓		✓
Goel	2019	66.0/66.5	200	100/99	300/225 mm Hg	71.6	✓			✓

*T* tourniquet, *N T* non- tourniquet, *DVT* deep venous thrombosis, *NA* not available, *K-C* knee circumference, *D-d* D-dimers, *SBP* systolic blood pressure

### Quality assessment

The quality assessment of the primary studies is summarized in Table 2. The Jadad scores ranged from 0 to 7. Thirteen of fifteen studies had a Jadad score of at least 4 and thus were considered high quality. Two studies, with a Jadad score of less than 3, were deemed low quality and ultimately excluded.

**Table 2:** Quality assessment of RCTs included in the review

Study	Explicit eligibility criteria	Similarity of baseline characteristics	Revised Jadad score				
			Random sequence generation	Allocation concealment	Double-blind	Withdrawals and dropouts	Sum (4–7 high quality)
Abdelsalam and Eyres [30]	Yes	Yes	2	1	2	0	5
Wakankar et al. [29]	Yes	Yes	2	1	0	1	4
Aglietti et al. [28]	Yes	Yes	2	1	0	1	4
Vandenbussche et al. [27]	Yes	Yes	2	2	2	1	7
Wauke et al. [26]	Yes	Yes	2	1	0	1	4
Li et al. [31]	Yes	Yes	2	2	1	1	6
Zhang et al. [25]	Yes	Yes	2	2	0	1	5
Zhou et al. [18]	Yes	Yes	0	0	0	1	1
Yin et al. [17]	Yes	Yes	1	1	0	1	3
Ejaz et al. [24]	Yes	Yes	2	2	0	1	5
Mori et al. [23]	Yes	Yes	2	1	0	1	4
Zhang et al. [22]	Yes	Yes	2	0	1	1	4
Zhou et al .[21]	Yes	Yes	2	2	0	1	5
Wu et al. [20]	Yes	Yes	2	2	2	1	7
Goel et al. [19]	Yes	Yes	2	2	2	1	7

### Effects on DVT

**Fig. 2:**
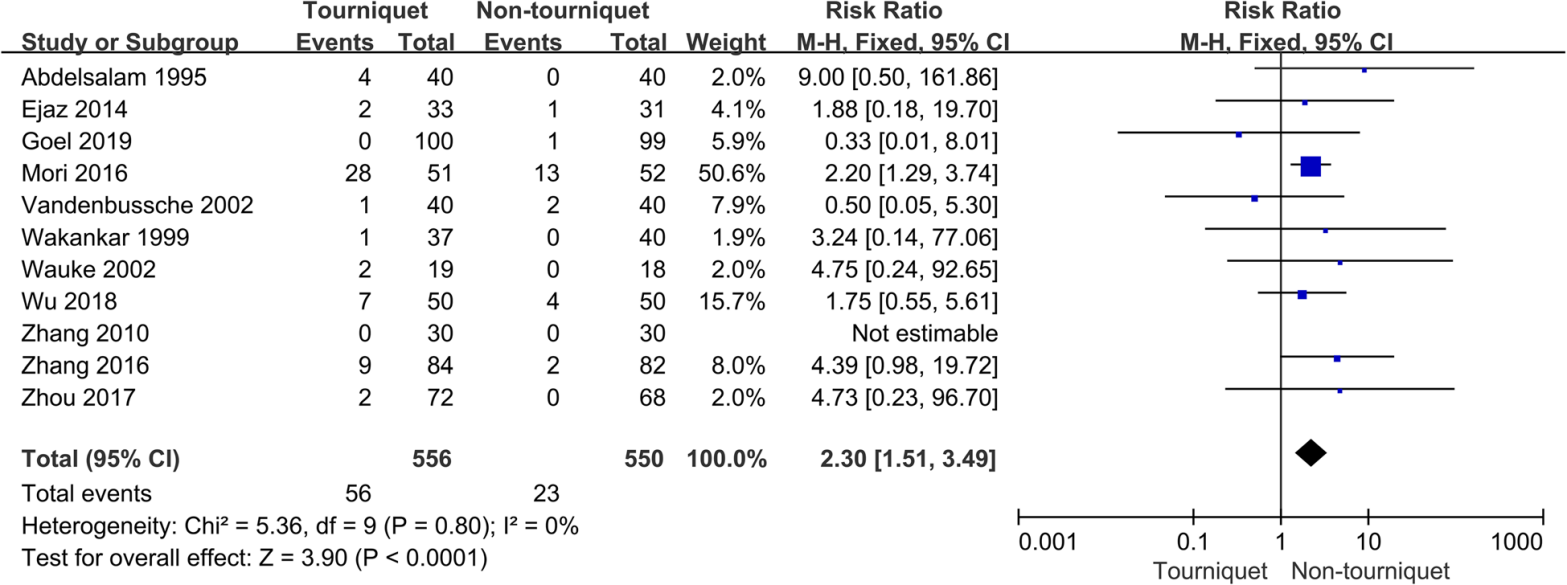
Occurrence of a deep vein thrombosis

Based on the inclusion criteria, a total of 11 trials with 1106 patients provided data of DVT were included in the study (Fig. 2). There was a significant statistical difference in DVT between the tourniquet and non-tourniquet groups (RR 2.30, 95% CI 1.51–3.49, *P* < 0.0001, *I*^2^ = 0%).

### Effects on knee circumference

**Fig. 3:**
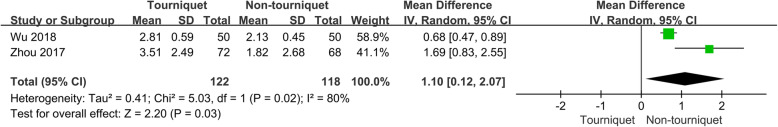
Knee circumference difference, 1st day postop

**Fig. 4:**
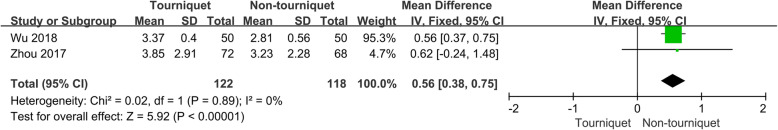
Knee circumference difference, 3rd day postop

**Fig. 5:**
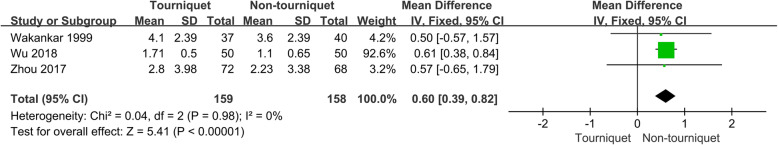
Knee circumference difference, 5th–7th day postop

**Fig. 6:**
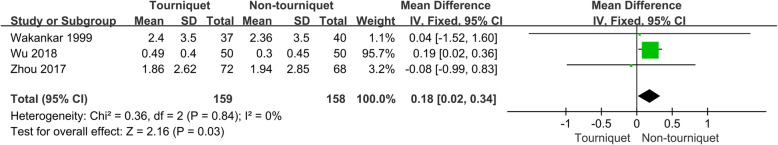
Knee circumference difference, 3rd–6th week postop

**Fig. 7:**
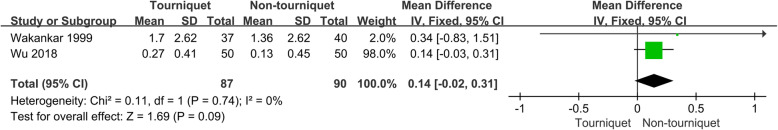
Knee circumference difference, 4th–6th month postop

Four trials provided data of knee circumference, and three of these studies with 317 patients were included in analysis. The meta-analysis showed that the tourniquet group had a significantly larger knee circumference difference than the non-tourniquet group in the 1st, 3rd, and from the 5th to the 21st days, and from the 3rd to the 6th weeks after surgery (*P* < 0.05) (Figs. 3, 4, 5, and 6). However, 4 to 6 months after the surgery, no significant difference in knee circumference difference was found between the two groups (MD 0.14, 95% CI −0.02–0.31, *P* = 0.09, *I*^2^ = 0%) (Fig. 7).

### Effects on D-dimers

Three trials provided data of D-dimers, but only one study with 20 patients was included because the two other trials had a revised Jadad scale score of less than 3 and were ruled out. Therefore, a meta-analysis of D-d dimers was not possible.

### Effects on pain

**Fig. 8:**
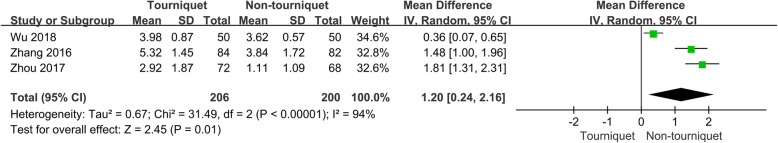
Pain, 3rd day postop

**Fig. 9:**
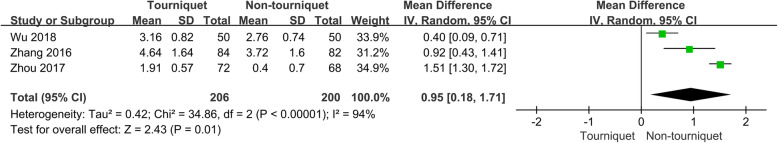
Pain, 5th day postop

**Fig. 10:**
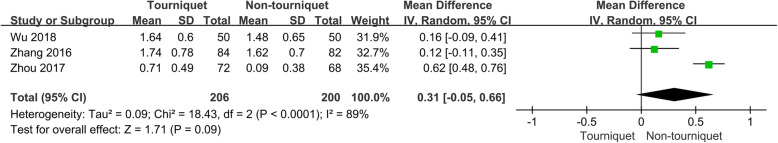
Pain, 3rd week–1st month postop

Eight trials provided data of pain score measured using the visual analog scale (VAS). Three of these studies with 406 patients were included in the analysis. The results show that the VAS score in the tourniquet group was higher than in the non-tourniquet group on the 3rd and 5th days after surgery (*P* < 0.05) (Figs. 8 and 9). However, there was no significant difference in VAS score between the two groups (MD 0.31, 95% CI −0.05–0.66, *P* = 0.09, *I*^2^ = 89%) (Fig. 10).

### Publication bias

**Fig. 11:**
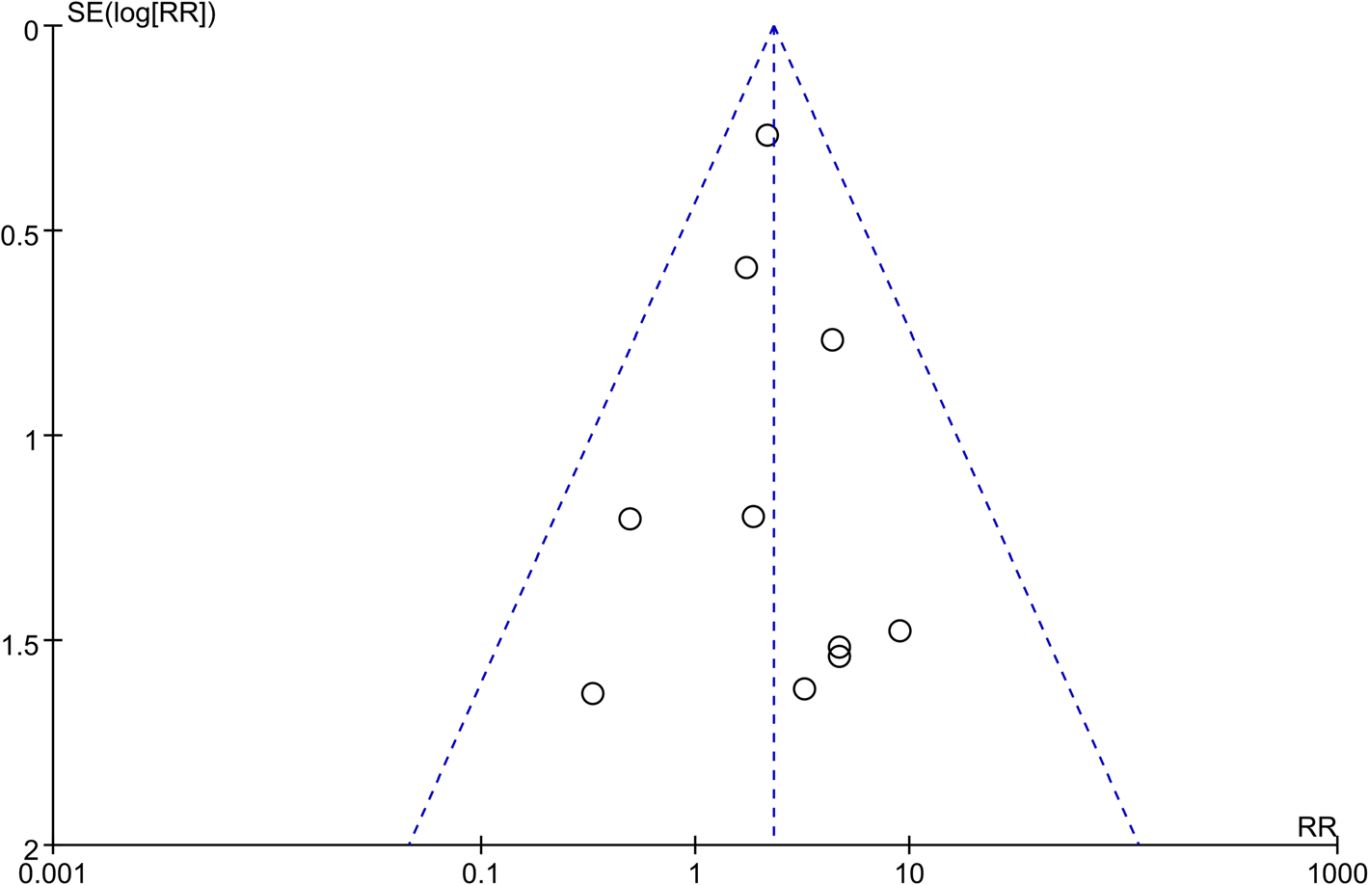
Funnel plot of DVT

In this study, with DVT after TKA as the main observation index, publication bias was analyzed in 11 studies. All the studies were distributed symmetrically with a small top and a large bottom indicating no significant publication bias in the 11 included studies (Fig. 11).

## Discussion

Tourniquets are widely used in various surgical procedures, but their risks and benefits are still highly controversial. The use of tourniquets achieves a bloodless field for visualization and cement interdigitation [5], but this also gradually exposes its potential disadvantages. A number of reports have studied the effects of tourniquets on the risk of postoperative thrombosis, but their inclusion criteria, operative skill of the surgeons, and number of cases all varied, resulting in varying outcomes as well. Therefore, we included these existing data in the meta-analysis to provide an objective basis for clinical practice. The results showed that tourniquet use in TKA increases the incidence of postoperative DVT and exacerbated knee pain and swelling in the early postoperative period. However, in terms of long-term effects, there were no significant differences in knee swelling and pain between the two groups.

Thrombosis is one of the most common and dangerous postoperative complications. Venous stasis, endothelial injury, and hypercoagulability are the main factors of thrombosis according to Virchow’s triad [32]; this holds true for patients undergoing TKA. Majority of patients receiving TKA are elderly with vascular aging and marked changes in hemorheology accompanied by varying degrees of hypercoagulability. All these factors put the patient’s blood in a state of high coagulation. High pressure and prolonged ligation lead to limb ischemia, and when circulation resumes, ischemia reperfusion leads to secondary injury of endothelial cells [21], temporarily increased blood volume and systemic vascular resistance, induction of hypercoagulability, and activation of fibrinolytic activity [33]. Endothelial cells and skeletal muscles injured by this mechanism of ischemia-reperfusion produce more oxygen free radicals and release inflammatory mediators to promote oxidative stress levels and the inflammatory reaction [33]. Inflammation stimulates thrombosis, which in turn promotes inflammation; these two processes are interdependent and mutually reinforce each other [34]. These mechanisms suggest that tourniquet use may increase the incidence of thrombi, which is consistent with our conclusions. However, the effect of tourniquet on thrombus development remains controversial because many studies have different conclusions. Fukuda et al. [35] showed that tourniquet use in TKA did not increase the incidence of thrombus postoperatively, and the difference between the two groups was not statistically significant. Yi et al. [11] and Alcelik et al. [12] reached the same conclusion. This difference may be related to the inclusion criteria because we included only high-quality literature on unilateral knee replacement.

D-dimers are the degradation product of fibrinolytic enzymes on cross-linked fibrin, indicating the development of thrombus. A D-dimer test could reliably exclude DVT with a negative predictive value of 99% [36]. Since only one study on D-dimers was included, no effective analysis could be performed, but this study also showed that the use of tourniquets increased D-dimer levels [28]. In a similar study, Reikeras et al. [37] found that D-dimer levels increased immediately after tourniquet release. This suggested that tourniquet use in TKA might affect coagulation function and promote thrombosis. D-dimers are affected by many factors, such as surgery, trauma, bleeding, pregnancy, and tumors. As mentioned above, patients may preoperatively have hypercoagulability and hyperfibrinolysis, so elevation of D-dimer is not an independent risk factor for thrombosis after TKA.

Pain and swelling of the knee joint are common symptoms after TKA and can also accompany DVT. Our analysis shows that the use of a tourniquet aggravated postoperative pain and swelling in the short term. Postoperative pain stimulation will lead to sympathetic excitement and a stress response, leading to endocrine disorders and aggravating hypercoagulability. Endothelial cell damage caused by ischemia-reperfusion can aggravate pain, and reactive hyperemia of the affected limb can aggravate swelling [38].

Although our results endorse the absence of tourniquets in TKA, we cannot conclude this by evaluating these indicators alone. The duration of tourniquet use, tourniquet pressure, and operation time all affect the prognosis. Clinicians often use tourniquets based on their own experience to determine the use of time and stress, regardless of the patient’s baseline blood pressure. All of these can have a big impact on the clinical outcome. The use of tourniquets should be considered in combination with other beneficial treatment modalities.

## Conclusions

This meta-analysis of randomized placebo-controlled clinical trials suggests that tourniquet application could increase the incidence of postoperative DVT and aggravate postoperative pain and swelling in the short term. Our results indicate that TKA without tourniquet use is superior to TKA with tourniquet use when evaluating postoperative thrombosis, pain, and swelling. However, only a few cases were studied, and the study time span was long; because of this, the application of tourniquets in TKA should not be completely stopped. Therefore, this paper only serves as a clinical reference, which needs to be confirmed by more prospective studies. It is necessary to combine relevant clinical indicators and the experience of the surgeon to decide whether to apply a tourniquet in TKA.

## Data Availability

All data generated or analyzed during this study are included in published articles.

## Abbreviations

TKA: Total knee arthroplasty
TKR: Total knee replacement
RCTs: Randomized controlled trials
VAS: Visual analog scale
DVT: Deep venous thrombosis
K-C: Knee circumference
D-d: D-dimers
SBP: Systolic blood pressure
PRISMA: Preferred Reporting Items for Systematic Reviews and Meta-Analyses
MD: Mean difference
CI: Confidence interval
RR: Relative risk
